# Factors associated with climate change worry in older adults: a multidimensional health perspective

**DOI:** 10.1186/s12877-026-07394-x

**Published:** 2026-03-28

**Authors:** Birol Önal, Ayşe Abit Kocaman, Seyma Nur Onal

**Affiliations:** 1 Department of Physiotherapy and Rehabilitation, Atatürk University, Erzurum, Türkiye; 2 Department of Physiotherapy and Rehabilitation, Kırıkkale University, Kırıkkale, Türkiye; 3 Vocational School of Health Services, Physiotherapy Program, Bartın University, Bartın, Türkiye

**Keywords:** Climate change worry, Older adults, Urban–rural residence, Sleep quality, Education

## Abstract

**Background:**

Climate change worry is increasingly recognized as a significant psychological response to environmental threats; however, research in older adults has largely focused on climate-related anxiety or isolated health outcomes. Studies examining climate change worry from a multidimensional health perspective are limited, particularly among older adults living in communities. This study aimed to investigate the relationships between climate change worry and multidimensional health characteristics in older adults.

**Methods:**

A cross-sectional study included 148 community-dwelling older adults in Türkiye. Participants were assessed using the Climate Change Worry Scale, Standardized Mini Mental State Examination (SMMSE), Athens Insomnia Scale (AIS), World Health Organization Well-Being Index (WHO-5), Geriatric Depression Scale–15 items (GDS-15), and International Physical Activity Questionnaire–Short Form (IPAQ-SF). Multiple linear regression analyses were performed to examine factors associated with climate change worry.

**Results:**

The overall regression model was statistically significant (R² = 0.221, F (9,138) = 4.36, *p* < 0.001). Higher AIS scores (β = 0.279, *p* = 0.004), higher education level (β = 0.193, *p* = 0.027), and living in urban areas (β =-0.173, *p* = 0.039) were significantly associated with greater climate change worry. Age was negatively associated with climate change worry (β =-0.164, *p* = 0.044), indicating lower worry in older participants. SMMSE, WHO-5, GDS-15, IPAQ-SF, and sex were not significantly associated with climate change worry in the present model.

**Conclusions:**

Among community-dwelling older adults, climate change worry was associated with specific sociodemographic and lifestyle-related factors, including sleep quality, educational level, urban residence, and age. In the present model, cognitive status, general well-being, depressive symptoms, and physical activity were not significantly associated with climate change worry. These findings support the need for further research on climate-related concern in later life, particularly in relation to contextual factors.

## Introduction

Climate change is a global emergency that is already exerting devastating effects on both our planet and human health [1]. The World Health Organization (WHO) identifies climate change as ‘the greatest public health threat of the 21st century [2]. The health impacts of climate change are expected to increase exponentially in the coming years if no preventive measures are taken [3, 4]. A study incorporating data from multiple countries indicates that climate change is responsible for an additional 400,000 deaths annually, with projections suggesting this number could rise to 700,000 deaths per year by 2030. Populations in all countries, regardless of altitude or income level, are threatened by climate change, irrespective of their age or socioeconomic status [5].

Older adults are particularly affected by climate change due to their physiological vulnerabilities, burden of chronic diseases, and limited capacity to adapt to environmental changes [6, 7]. Structural and functional changes in organ systems associated with aging render individuals more vulnerable to external environmental stressors; reduced thermoregulatory capacity, weakened immune function, and cardiovascular insufficiencies increase the risks associated with climate change. Rising temperatures, in particular, significantly elevate mortality among older adults with cardiovascular, respiratory, and renal diseases [8]. Furthermore, air pollution exacerbates the prevalence of chronic diseases that increase with age, adversely affecting quality of life [9, 10]. Extreme weather events resulting from climate change significantly affect not only physical health but also the mental health of older adults. Following events such as floods, storms, and prolonged heatwaves, increases in post-traumatic stress disorder, anxiety, and depression have been observed, with these effects being particularly pronounced among older individuals [11, 12].

In addition to individual health-related vulnerabilities, the living environment plays a crucial role in shaping older adults’ perceptions and psychological responses to climate change. Differences between urban and rural settings may influence exposure to climate-related risks, access to health services, availability of social support, and access to information related to environmental threats [13–15]. Older adults living in urban areas are more likely to be exposed to air pollution, urban heat island effects, and dense media coverage of climate change, factors that have been associated with heightened environmental awareness and increased climate-related worry and psychological distress [13, 16]. In contrast, rural older adults may experience stronger place attachment, different patterns of risk perception, and more limited access to climate-related information and health resources, which may shape their level of concern through distinct psychological and contextual pathways [17, 18]. Overall, existing evidence suggests that climate-related psychological outcomes among older adults are strongly shaped by contextual factors, and neither urban nor rural environments can be considered uniformly protective [7, 14]. Accordingly, the present study considers area of residence as an important contextual variable when examining climate change worry among older adults.

It is evident that older adults face multidimensional vulnerabilities as a result of climate change [19]. Physical health problems, psychological effects, and socioeconomic inequalities render older adults more vulnerable to the adverse consequences of climate change, with psychological impacts increasingly receiving attention in the literature [20]. Older adults may experience a range of adverse health outcomes, such as depression, sleep disturbances, and reduced quality of life, due to feelings of anxiety, fear, helplessness, and hopelessness regarding the future impacts of climate change [21]. The majority of studies focusing on older adults in the context of climate change have primarily addressed climate-related anxiety or eco-anxiety, whereas research specifically examining climate change worry remains limited. Although some studies have explored associations between climate change worry and selected health indicators in older adults, there is a notable lack of research systematically and comprehensively investigating the relationship between climate change worry and concrete, measurable health components, such as physical activity levels, sleep quality, cognitive functioning, and psychological well-being. In particular, studies adopting a multidimensional health perspective to examine climate change worry among older adults are scarce, and to our knowledge, no such study has been conducted in Türkiye. The overall aim of this study is to examine climate change worry among older adults within a multidimensional health perspective by investigating its associations with physical, psychological, cognitive, and sociodemographic factors. The specific objectives of the study are to: (i) determine the level of climate change worry among older adults; (ii) evaluate the relationships between climate change worry and physical activity level, sleep quality, cognitive functioning, and psychological well-being; and (iii) examine the effect of area of residence (urban versus rural) on climate change worry.

## Methods

### Participants and study design

This cross-sectional study was conducted among community-dwelling older adults in Erzurum and Kırıkkale, Türkiye. According to official population statistics, the number of individuals aged 65 years and older was 74,436 in Erzurum and 39,581 in Kırıkkale, indicating that both provinces provide an appropriate setting for the study [22]. Participants were recruited using a convenience sampling method from community-based public settings where older adults commonly gather, including parks, places of worship, and shopping centers. Data collection was conducted between September and November 2025. During the recruitment process, 200 older adults were approached, of whom 148 met the inclusion criteria and agreed to participate in the study. All assessments were administered face-to-face by the researchers in a single session. Completion of the demographic form and study questionnaires required approximately 30–35 min for each participant.

The study included individuals aged 65 years and older who were living in the community. Written informed consent was obtained from all participants prior to data collection. Inclusion criteria were: age ≥ 65 years, living in the community (not in institutional care), sufficient cognitive ability to respond to questionnaires, and a Standardized Mini-Mental State Examination (SMMSE) score of 24 or higher. Exclusion criteria included a diagnosis of dementia, severe psychiatric or neurological disorders, and communication difficulties or cognitive impairments that could interfere with reliable questionnaire completion. Ethical approval for the study was obtained from the Non-Interventional Ethics Committee of Atatürk University Faculty of Medicine (Approval number: 07/20). All procedures were conducted in accordance with the Declaration of Helsinki.

An a priori power analysis was performed using G*Power 3.1 software (Heinrich Heine University, Düsseldorf, Germany) to determine the required sample size for multiple linear regression analysis. Assuming a medium effect size (f² = 0.15), an alpha level of 0.05, a statistical power of 0.90, and nine predictors, the minimum required sample size was calculated as 141 participants.

### Data collection tools

A structured form developed by the researchers was used to collect participants’ demographic characteristics, including age, body mass index, gender, education level, marital status, area of residence (rural/urban), presence of chronic diseases, and medication use. Area of residence was classified based on the participants’ official place of residence as reported by the participants; urban residence was defined as living within city or district centers, whereas rural residence was defined as living in villages or settlements outside urban administrative boundaries. All assessment tools used in this study were previously translated, validated, and widely used in the Turkish population, and permission was obtained from the original authors where required.

#### Climate Change Worry Scale

It was used to assess participants’ levels of worry related to climate change [23]. The Turkish adapted version of this scale is a unidimensional instrument developed to measure climate change worry and consists of a total of 10 items [24]. Items are responded to using a five-point Likert-type frequency rating scale, and administration takes approximately 3–5 min. The minimum possible score is 10, while the maximum possible score is 50. The internal consistency of the Climate Change Worry Scale in the present sample was good (Cronbach’s α = 0.86).

#### Standardized Mini Mental State Examination

The Standardized Mini Mental Examination (SMMSE) was administered to assess participants’ cognitive abilities. This 30-item screening tool evaluates orientation, attention, memory, language, and visuospatial functions and is a standardized version adapted and validated for the Turkish population [25]. The administration of the test takes approximately 5–15 min and it is widely used in both clinical and field studies. The maximum possible score is 30, and scores below 24 are generally considered indicative of cognitive impairment [26].

#### Geriatric Depression Scale

The 15-item short form of the Geriatric Depression Scale (GDS-15) was administered to assess participants’ depressive symptoms. Specifically developed for older adults, this scale features a simple ‘yes/no’ response format and takes approximately 5–10 min to complete. The GDS-15 is widely used in both clinical and community-based screenings and is preferred for its ability to provide reliable information in a short time [27, 28].

#### Athens Insomnia Scale

The Athens Insomnia Scale (AIS) was administered to assess participants’ sleep quality and insomnia symptoms. This self-report scale, based on the International Classification of Diseases-10 (ICD-10) criteria, consists of a total of eight items, five related to nighttime sleep (e.g., sleep onset, night awakenings) and three related to daytime functioning. Each item is scored from 0 to 3, with total scores ranging from 0 to 24. Scores of 6 and above are generally considered indicative of a risk for insomnia [29].

#### International Physical Activity Questionnaire – Short Form

The International Physical Activity Questionnaire – Short Form (IPAQ-SF) was administered to assess participants’ levels of physical activity. This self-report questionnaire collects information on walking, moderate and vigorous physical activities, as well as sedentary behavior, over the past seven days [30]. The IPAQ-SF is widely used to categorize physical activity levels in adults and is preferred in clinical and field studies due to its ease of administration and low cost.

#### World Health Organization Well-Being Index

The World Health Organization Well-Being Index (WHO-5) was used to assess participants’ psychological well-being. The original version of this brief scale consists of five items and was adapted into Turkish in 1999 [31]. The scale assesses the extent to which participants have felt cheerful, calm, energetic, rested, and satisfied with daily life over the past 14 days. Each item is scored from 0 (never) to 5 (always), with total raw scores ranging from 0 to 25. The scores can also be converted to a 100-point index by multiplying the raw score by four [31, 32].

### Statistical analysis

Data were analyzed using SPSS Statistics for Windows, Version 25.0 (IBM Corp., Armonk, NY, USA). The normality of continuous variables was assessed using both visual methods (histograms, probability plots) and analytical methods (Kolmogorov-Smirnov and/or Shapiro-Wilk tests). Descriptive statistics for normally distributed continuous variables were expressed as mean ± standard deviation (SD), while non-normally distributed variables were presented as median and interquartile range (IQR). Categorical variables were reported as counts (n) and percentages (%). Comparisons of data among older adults were conducted using the independent samples t-test for normally distributed variables and the Mann-Whitney U test for non-normally distributed variables. Multiple linear regression analysis was performed to investigate factors associated with climate change worry, and variables were included in the model based on their theoretical relevance, including sociodemographic characteristics, psychological status, lifestyle factors, and cognitive function; cognitive function, assessed using the SMMSE, was included because the perception and appraisal of climate-related risks may partly depend on cognitive abilities even among community-dwelling older adults without severe impairment. Prior to modeling, regression assumptions were assessed, including normality, homoscedasticity, and independence of residuals. Multicollinearity was assessed using variance inflation factors (VIF) and tolerance values; no evidence of problematic multicollinearity was observed, with all VIF values below 2. A p-value of < 0.05 was considered statistically significant.

## Results

A total of 148 participants were included in the study. Demographic and clinical characteristics of the participants are presented in Table 1. The mean age of the participants was 70.5 (5.6) years. The mean body mass index (BMI) was 29.1 (5.0). The SMMSE score was 24.9 (4.9). Of the participants, 54.7% lived in urban areas and 45.3% lived in rural areas. Regarding education level, 10.8% were illiterate, 64.9% had completed primary school, 14.9% secondary school, 4.7% high school, and 4.7% university. In terms of gender distribution, 59.5% were women and 40.5% were men. Additionally, 88.5% of the participants had at least one chronic disease, and 89.9% reported regular medication use.

**Table 1:** Sociodemographic and clinical characteristics of the participants

	Mean (SD)
Age (years)	70.5 (5.6)
Body Mass Index (kg/m²)	29.1 (5.0)
Standardized Mini Mental State Examination (0–30)	24.9 (4.9)
WHO-5	12.1 (4.9)
GDS-15	7.4 (1.9)
IPAQ-SF	968.9 (1421.8)
	*n* (%)
Sex	
Female	88 (59.5)
Male	60 (40.5)
Place of Residence	
Urban	81 (54.7)
Rural	67 (45.3)
Education Level	
Illaterate	16 (10.8)
Primary School	96 (64.9)
Middle School	22 (14.9)
High School	7 (4.7)
University	7 (4.7)
Presence of Chronic Disease	
At least one chronic disease	131 (88.5)
No chronic disease	17 (11.5)
Medication Use	
Yes	133 (89.9)
No	15 (10.1)

Comparisons of the mean scores according to demographic variables are presented in Table 2. There was a significant difference between participants living in urban and rural areas (t = 3.07, *p* = 0.003). No significant difference was found between male and female participants (t = 1.04, *p* = 0.297). According to education level, a borderline significant difference was observed among the groups (F = 2.43, *p* = 0.050).

**Table 2:** Comparisons of climate change worry scores by sociodemographic characteristics

		*n*	Mean (SD)	t/F	p
Place of Residence	Urban	81	32.57 (5.72)	3.07 (t)	0.003
	Rural	67	29.33 (7.13)		
Sex	Female	88	31.56 (6.11)	1.04 (t)	0.297
	Male	60	30.41 (7.18)		
Education Level	Illaterate	16	30.44 (7.49)	2.43 (F)	0.050
	Primary School	96	30.58 (6.35)		
	Middle School	22	30.59 (6.11)		
	High School	7	36.29 (7.16)		
	University	7	36.14 (6.57)		

Multiple linear regression analysis was conducted to examine factors associated with climate change worry (Table 3). The overall model was statistically significant (R² = 0.221, F (9,138) = 4.359, *p* < 0.001), indicating that approximately 22% of the variance in climate change worry was explained by the independent variables included in the model.

**Table 3:** Multiple linear regression analysis of factors associated with climate change worry

	B	SE β		t	*p*	%95 CI
Constant	36.044	8.414	-	4.284	< 0.001	[19.408, 52.680]
SMMSE	0.151	0.122	0.112	1.237	0.218	[-0.090, 0.392]
AIS	0.421	0.144	0.279	2.927	**0.004**	[0.136, 0.705]
WHO-5	0.020	0.121	0.015	0.166	0.868	[-0.219, 0.260]
GDS-15	0.165	0.299	0.049	0.551	0.582	[-0.427, 0.757]
Age	-0.196	0.096	-0.164	-2.036	**0.044**	[-0.387,-0.006]
IPAQ	0.001	0.000	0.123	1.577	0.117	[0.000, 0.001]
Sex (female = ref)	-0.725	1.133	-0.054	-0.640	0.523	[-2.966, 1.516]
Place of Residence (rural = ref)	-2.280	1.094	-0.173	-2.083	**0.039**	[-4.444, -0.116]
Education Level	1.421	0.637	0.193	2.232	**0.027**	[0.162, 2.679]

*Abbrevations*: *SMMSE* Standardized Mini Mental State Examination, *AIS* Athens Insomnia Scale, *WHO-5* World Health Organization-Five Well-Being Index, *GDS-15*: Geriatric Depression Scale, *IPAQ-SF* International Physical Activity Questionnaire – Short Form

Values in bold represent statistically significant results (*p* < 0.05)

*R* = 0.447, R² = 0.221, F (9,138) = 4.359, *p* < 0.001

The analysis revealed that higher Athens Insomnia Scale (AIS) scores were positively associated with climate change worry (β = 0.279, *p* = 0.004), suggesting that participants with poorer sleep quality reported higher levels of worry. Age was negatively associated with climate change worry (β =-0.164, *p* = 0.044), indicating that younger participants tended to have higher worry scores. Living area was also significantly associated with climate change worry (β =-0.173, *p* = 0.039), with individuals living in urban areas reporting higher levels of worry than those living in rural areas. Additionally, education level showed a positive association (β = 0.193, *p* = 0.027), with participants who had higher levels of education reporting greater climate change worry. In contrast, SMMSE score, WHO-5 well-being index, GDS-15 depression score, physical activity level (IPAQ), and gender were not significantly associated with climate change worry. (all *p* > 0.05).

## Discussion

This study provides a comprehensive examination of the associations between climate change worry and multiple health-related variables, including physical activity, sleep quality, and cognitive status, in community-dwelling older adults. It also provides evidence on factors associated with climate change worry in Türkiye. The findings indicate that increasing age was associated with lower levels of climate change worry, whereas higher educational attainment, poorer sleep quality, and urban residence were associated with higher levels of worry. In the present model, cognitive status, physical activity level, overall psychological well-being, and depressive symptoms were not significantly associated with climate change worry.

The literature indicates that individuals living in urban areas tend to exhibit higher levels of climate change-related worry and concern [33, 34]. The number of studies examining climate change worry levels among older adults living in rural versus urban areas is limited. Our findings suggest that urban residence was associated with higher climate change worry in the present sample. Several contextual factors may help explain this finding. First, the concept of ‘place attachment’ is an important factor shaping perceived threats of climate change among older adults living in rural areas. Rural residents tend to have strong and close connections with the land and nature. Additionally, these strong bonds may lead rural individuals to underestimate or overlook environmental threats [35]. Second, awareness of and access to information about climate change are more limited in rural areas compared to urban areas. Individuals living in urban regions can more easily access information about climate change through media, educational institutions, and social networks, and therefore perceive the climate crisis as a more serious threat. In rural areas, lower education levels and limited access to information sources are among the main factors negatively affecting climate change awareness [34, 35]. These findings are consistent with previous studies conducted in several European countries and the United Kingdom, where urban residents tend to report higher levels of climate change concern compared to those living in rural areas. Large-scale cross-national studies from Europe suggest that rural residence is often associated with higher climate scepticism and lower perceived climate-related risk, even after controlling for education and income [35, 36]. However, this pattern is not universal. Evidence from distinct climatic and socioeconomic contexts, such as Pakistan and rural Australia, reveals higher climate concern among rural populations, likely driven by lived climate experiences and direct vulnerability [34, 37]. Collectively, this suggests that urban–rural differences in climate worry are highly context-dependent, shaped by a complex interplay of environmental exposure, socioeconomic conditions, and cultural factors [38, 39].

A further important finding of this study was that poorer sleep quality was significantly associated with higher climate change worry in older adults. This is consistent with the broader literature linking sleep disturbances with anxiety-related responses, including climate-related distress [40]. Previous research supports this association; for instance, sleep quality was found to explain 7% of the variance in climate anxiety among older adults [41], and similar associations have been documented in younger populations [42]. One possible explanation is that insufficient sleep may be associated with reduced emotional regulation capacity, which could heighten responses to complex and uncontrollable threats such as climate change [43]. Consistent with our findings, evidence from diverse geographical and climatic contexts suggests an association between climate-related worry and sleep disturbances. Large-scale cross-national studies across Europe and North America have consistently shown that heightened eco-anxiety is linked to insomnia symptoms and poorer mental health [42, 44, 45]. Importantly, evidence from Türkiye further supports this global pattern, with recent studies among older adults demonstrating a significant positive association between climate change anxiety and insomnia severity [41]. In addition, research from distinct climatic regions suggests that environmental exposures such as rising nighttime temperatures, heatwaves, and climate-related disasters are associated with shorter and more fragmented sleep [46–48]. Global datasets indicate that older adults are disproportionately vulnerable to this heat-related sleep loss [47, 48]. Taken together, these findings suggest that sleep quality may be a relevant factor when examining climate-related worry in older adults.

Our findings suggest that education level was associated with climate change worry among older adults. Higher educational attainment is associated with greater access to scientific knowledge, which may facilitate a more nuanced understanding of complex environmental processes and their potential consequences [49]. Consistent with evidence from diverse international contexts, individuals with higher levels of education tend to perceive climate change as a more serious and imminent threat [33, 50, 51]. The present results suggest that education is related to how climate-related risks are understood and appraised in later life. Evidence from diverse cultural and socioeconomic settings supports this interpretation. In Western contexts such as Canada and the United Kingdom, older adults often report a strong sense of intergenerational responsibility; however, engagement with climate issues may be limited by complex or inconsistent information, a challenge that higher educational background may help to overcome [52–55]. Similarly, studies from regions with different climatic and socioeconomic conditions, including Egypt and Israel, indicate that climate literacy and environmental self-efficacy, factors closely linked to education, are associated with stronger pro-environmental attitudes and greater climate-related concern [56, 57]. These findings suggest that education may function as a contextual factor related to how climate-related risks are understood and appraised among older adults [58]. The educational distribution in the present sample is broadly consistent with national statistics for older adults in Türkiye, in which primary-level education is the most common and secondary or higher education remains less frequent. Nevertheless, the relatively small number of participants with high school and university education should be taken into account when interpreting the association between education level and climate change worry.

Previous research indicates that climate-related anxiety and worry vary across the life course rather than following a simple linear age pattern. While adolescents and young adults generally report higher levels of climate-related anxiety, worry, and negative emotional responses [59, 60], evidence from multinational studies suggests that even within younger age cohorts, older individuals may experience greater levels of worry [59]. Large population-based surveys encompassing broader age ranges further demonstrate that older adults can report levels of climate-related worry comparable to, or in some cases exceeding, those observed in younger populations, depending on how climate-related emotional responses are defined and measured [61]. European studies additionally emphasize that age effects often become attenuated or change direction after accounting for sociodemographic and psychological factors, highlighting the non-linear nature of age-related differences in climate change worry [62]. Importantly, older adults do experience climate-related worry, although its expression may differ from that of younger groups. For example, research conducted in Italy shows that a substantial proportion of individuals aged over 41 express pronounced concern regarding the impacts of climate change on future generations [63]. At the same time, studies among children and adolescents indicate that although climate-related knowledge tends to increase with age, younger individuals may still report higher levels of concern and a stronger willingness to engage in climate action [64]. Taken together, these findings suggest that age-related differences in climate change worry may reflect variations in future orientation, emotional regulation, and life-course experiences rather than chronological age alone. Consistent with this perspective, the present study found that climate change worry decreased with increasing age within the older adult population, further supporting the heterogeneity of climate-related emotional responses in later life.

Although the Climate Change Worry Scale has been validated as a unidimensional instrument, the content of its items appears to encompass different aspects of climate-related concern, including future-oriented worries, concerns about current environmental degradation, perceived impacts on oneself or family, and the frequency of climate-related thoughts. Therefore, the total score used in the present study should be interpreted as reflecting overall climate change worry rather than distinct subtypes of concern. It is possible that factors such as education level or urban residence may be more strongly related to some aspects of climate-related worry than others; however, the present sample size did not allow for a more detailed item-level analysis. Future studies with larger samples may help clarify which aspects of climate-related worry are most salient in older adults.

Our findings indicate that overall psychological well-being (WHO-5), depressive symptoms, cognitive status, and physical activity level were not significantly associated with climate change worry in the present model. However, these non-significant findings should not be interpreted as evidence that climate change worry is conceptually independent from broader mental health or behavioral factors. Rather, they suggest that no significant associations were observed for these variables within the current sample and model. It is possible that climate change worry may also be shaped by other contextual and psychosocial factors, such as individual values, sociocultural environment, media exposure, and direct experiences related to climate change.

### Limitations

Several limitations of this study should be acknowledged. First, key variables such as climate change worry, sleep quality, depression, and general well-being were assessed using self-report measures, which may be subject to response bias. Second, the sample included only community-dwelling older adults, excluding individuals living in nursing homes or those with severe cognitive impairment or serious health conditions, which may limit the generalizability of the findings to more vulnerable populations. In addition, the cross-sectional design precludes causal inferences between climate change worry and the associated multidimensional health factors. Finally, unmeasured contextual factors such as media exposure, personal climate-related experiences, and environmental beliefs may have influenced climate change worry and should be addressed in future longitudinal research.

## Conclusion

This study suggests that climate change worry in older adults is associated with selected sociodemographic and lifestyle-related factors. Within this sample, younger age, higher educational level, urban residence, and poorer sleep quality were associated with higher levels of climate change worry, whereas general well-being, depressive symptoms, and cognitive status were not significantly associated with climate change worry in the present model. By examining climate change worry from a multidimensional health perspective, this study highlights the importance of context-sensitive approaches in understanding climate-related concern among older adults. Future longitudinal and mixed-method studies incorporating broader contextual factors, such as media exposure and direct climate-related experiences, may further clarify these associations.

## Data Availability

The data that support the findings of this study are available from the corresponding author, [B.Ö], upon reasonable request.
